# Alone Together: Computer-Mediated Communication in Leisure Time During and After the COVID-19 Pandemic

**DOI:** 10.3389/fpsyg.2021.666655

**Published:** 2021-06-21

**Authors:** Jennifer V. Meier, Josephine A. Noel, Kai Kaspar

**Affiliations:** Department of Psychology, University of Cologne, Cologne, Germany

**Keywords:** COVID-19, uses and gratification approach, computer-mediated communication, leisure activities, personality, big five, inter-individual differences, perceived social closeness

## Abstract

In spring 2020, the COVID-19 outbreak was declared a global pandemic and several lockdowns were followed in Germany. To weaken the spread of the virus, SARS-CoV-2, people were required to reduce their face-to-face contact with others. Computer-mediated communication (CMC) offers opportunities to stay in touch with important people and still meets social needs. During the first lockdown in spring 2020, we collected data from 679 participants to explore the role of CMC for social interaction in the context of leisure activities and how personal characteristics contribute to such media use. Results showed that people complied with the restriction and shifted their communication to several media, especially instant messengers and video calls. Many leisure activities were performed *via* CMC, especially low-key activities, such as just talking or simply spending time together. Perceived social closeness to others was positively related to the quality and quantity of CMC. The extent of leisure activities that people experienced with others *via* communication media was higher among younger individuals, males, and individuals with higher scores on positive state affect, extraversion, openness, and fear of missing out. The preference for solitude showed a negative relation. The motivation to continue using media for communication after the pandemic was strongly related to the quality and quantity of actual use. Low extraversion and high openness were related to higher motivation for future use. Implications such as the importance of providing fast internet and good usability of communication media as well as the relevance of increasing media literacy of people are discussed.

## Introduction

In March 2020, the WHO has declared the outbreak of COVID-19 a global pandemic (Cucinotta and Vanelli, 2020). A few weeks later (March 22, 2020), a nationwide lockdown in Germany resulted, including several restrictions that aimed at reducing the spread of the virus. One crucial restriction was the reduction of contacts with other people outside the own household to an absolute minimum (Bundesregierung, 2020). Furthermore, restaurants were closed, celebrations and sports in groups were forbidden. All these restrictions had an enormous impact on individual freedom and the possibilities for leisure activities. In western societies, the perceived freedom is generally understood as the most important criterion for leisure experiences (Neulinger, 1974), and, hence, these restrictions presumably had a strong impact on leisure behavior that is based on interpersonal interaction. Interpersonal relationships are considered one of the most important aspects of human life (Argyle and Crossland, 1987), and spending time with others is a universal and basic need (Baumeister and Leary, 1995). Accordingly, people usually spend most of their time with others, and time spent alone is generally considered less pleasant than time spent with others (Larson et al., 1982). The current contact restrictions generally threaten the satisfaction of the need for social contacts. Therefore, alternative media-based ways of staying in contact are required.

According to the uses and gratification approach (U&G), people can satisfy some of their basic needs by using media. Although U&G is not a homogeneous theory (Rubin, 2009), different models of varying complexity share the assumption that recipients have several social and psychological needs that elicit specific expectations of how a medium can satisfy these needs. Research revealed that, in addition to the need for information, personal identity, and entertainment, the need for social interaction and integration is a central motive for media use (McQuail, 1994). Overall, the U&G is a useful theoretical framework emphasizing the active role of the individual user regarding media selection and consumption strategies to actively link need gratification with media choice and use (Katz et al., 1973). In this way, people may try to satisfy their need for social interaction and integration during contact restrictions by using computer-mediated communication (CMC). To investigate this critical aspect, the present study was conducted during the first nationwide lockdown in Germany in spring 2020. Based on the U&G approach, we pursued two objectives: On the one hand, we aimed to explore what communication media people use more frequently in the context of leisure activities during the first nationwide lockdown than before the pandemic, which specific leisure activities they conduct with others *via* CMC, and to what extent the perceived quality and quantity of CMC actually correlates with its primary goal of satisfying the need for social interaction and integration. On the other hand, recent U&G research has intensified the examination of inter-individual differences in media use. Tosun and Lajunen (2010) have already stated that people with different personal characteristics can differ in the needs they seek to satisfy *via* media use. Indeed, several studies in the field of U&G research showed that a wide range of individual characteristics of media users, such as age, gender, personality traits, and experienced affect, have a significant relationship with media use (e.g., Malik et al., 2016; Kaspar and Müller-Jensen, 2019; Kircaburun et al., 2020; Kaspar and Fuchs, 2021). Therefore, we also explored the role of personal characteristics in the context of leisure activities *via* CMC during the lockdown.

### Computer-Mediated Communication in Leisure Time During Contact Restrictions

To avoid losing contact with other people and to fulfill social needs, the digital world opens new possibilities to keep in touch with friends and family members (Saltzman et al., 2020). Without being in the same room and risking infection with SARS-CoV-2, the virus that causes COVID-19, CMC provides a safe way to spend time together with others. In fact, CMC was found to have a positive impact on well-being of people during contact restrictions (Nimrod, 2020), and spending time with family *via* media was associated with less depression (Ellis et al., 2020). Furthermore, parents reported that they and their children have increased their use of technology and social media since the contact restrictions (Drouin et al., 2020). These findings indicate that social interactions during the pandemic has shifted to the digital world. Hence, we formulated the following research question:

RQ1: What communication media are being used more frequently in the context of leisure activities during the first nationwide lockdown than before the pandemic?

Contact restrictions force people to try new ways to stay in touch with their family and friends and spend their leisure time together. For example, Perks (2020) found that the number of people playing online games has increased since COVID-19. In general, media is particularly useful for reinventing leisure activities (Meisner, 2020). Active and creative media users could transfer and adapt leisure activities to the digital world. Thus, we explored the following research question:

RQ2: What leisure activities do people experience with others *via* CMC during the lockdown?

In addition to the questions of which media were used and which activities were shifted to the digital world during the first lockdown, the question of satisfying the need for social contact and relationships remains. A central aspect of the need for social interaction and integration is social closeness (Aron et al., 2004). Perceived social closeness is not static but changes depending on the situation (Lee and Gillath, 2016). Consequently, the perceived social closeness with other people is likely to suffer greatly from contact restrictions. In this way, perceived social closeness in the context of CMC is an appropriate indicator of whether media use actually satisfies the social need for interaction and integration. According to the U&G approach, the gratification sought does not always correspond to the gratification received. Only if media use fulfills the expected gratification, it is likely that people will turn to this medium again if they have the same need (Palmgreen and Rayburn, 1982). The chosen media should, therefore, meet the expectations of social interaction and integration. In this context, Hecht (1978) defined communication satisfaction as one of the most important positive reinforcements, which is associated with the fulfillment of positive communicative expectations. In the present study, perceived satisfaction, thus, served as a quality indicator of CMC. In addition to perceived quality, communication intensity (i.e., quantity) may also be important. The more intensively media are used for communication purposes, the more likely respective needs will be sufficiently gratified at the end. However, we may assume that the intensity of CMC is associated with perceived satisfaction, because disappointing communication may lower motivation of one to use media intensively. Therefore, we explored the following research question during the lockdown:

RQ3: What is the relationship between the perceived quality of CMC in the context of leisure activities and the perceived social closeness to others, and is this relationship moderated by the quantity of CMC?

### Inter-Individual Differences in Media Use for Leisure Activities During Contact Restrictions

In the context of the U&G approach, the influential role of inter-individual differences in media user characteristics have been emphasized, including age, gender, affect, and diverse personality traits (e.g., Malik et al., 2016; Kaspar and Müller-Jensen, 2019; Kircaburun et al., 2020; Kaspar and Fuchs, 2021). However, research that examined the relationship between personal characteristics and media use during the current pandemic is sparse. Consequently, the present study focused on several personal characteristics that were found to be relevant in the time before the pandemic.

#### Age and Gender

The age of media users is related to their use of digital media for communication (Ono and Zavodny, 2003; Kimbrough et al., 2013; Özgüven and Mucan, 2013; Blackwell et al., 2017). Czaja et al. (2006) found that younger people adopted new technologies faster than older people did. Additionally, younger people used social media more often for communication purposes than older generations (Blackwell et al., 2017). The care of peer relations is thereby an important need (Kumar and Lim, 2008). In contrast to age, the relationship between gender and media use shows mixed results. Whereas some studies reported no gender differences in the intensity of media use (e.g., Özgüven and Mucan, 2013), other studies found that men used media more frequently and intensively than women did (e.g., Ono and Zavodny, 2003). However, other studies have shown that women, compared to men, preferred and more frequently used text messaging, social media, and online video calls (e.g., Kimbrough et al., 2013). Despite these mixed results, age and gender may play a significant role in CMC during a lockdown situation.

#### General Self-Efficacy

The COVID-19 pandemic is the fifth pandemic since the 1918 flu (Liu et al., 2020), but the first to affect European society of today. For most people in Germany, this is the first time they had to cope with the consequences of a pandemic. The ability of someone to overcome barriers and find new solutions in an unknown and difficult situation is conceptualized by self-efficacy theory of Bandura (1977). This theory postulates that the initiation and maintenance of behavior are determined by judgments and expectations about behavioral abilities and the likelihood of successfully dealing with the environmental challenges (Bandura, 1977). The theory also postulates that the same factors play an important role in psychological adjustment. Although the construct self-efficacy was originally defined as situation-specific, subsequent theories have described general self-efficacy as a cross-situational trait (Sherer et al., 1982; Shelton, 1990). Sherer et al. (1982) considered general self-efficacy as a personality trait with relative stability that influences the performance of an individual in certain situations, especially when they are novel. General self-efficacy differs in its expression between individuals to consider themselves capable of fulfilling task requirements (Schwarzer and Jerusalem, 1999; Chen et al., 2001). Schwarzer and Jerusalem (1999) assumed that deductive processes take place from general self-efficacy to specific self-efficacy expectations. This was shown by studies in which individuals with higher general self-efficacy exhibited higher computer self-efficacy as well as attributing more technological competencies to themselves (Paraskeva et al., 2008; McCoy, 2010). During the first lockdown, maintaining social contacts and leisure activities presented an unexpected new challenge that had to be overcome, and this challenge goes far beyond the mere application of technology. Hence, general self-efficacy can help to adapt and subsequently change the way leisure time is spent and social needs are met by using CMC. In addition, higher self-efficacy indicates better attitudes toward social media and explains the intention to use (Wang, 2015; Niu et al., 2021).

#### Positive and Negative State Affect

Another determinant of media use is mood. According to Mood Management Theory of Zillmann (1988), people select media content according to their mood to create or maintain a positive affective state. If people are in a bad mood or feel bored, they could choose entertaining media to lift their mood. Contact restrictions imposed by the government could create a bad mood. Aymerich-Franch (2020) explored this relationship and she showed that negative affect increased during the lockdown, whereas positive affect was lower than before. These changes in affect were significantly associated with an increase in (social) media use. Similarly, people might try to reach a positive mood by using CMC in the context of leisure activities.

#### Big Five Personality Traits

Inter-individual differences in personality are important when considering motivation and satisfaction in media use (Rubin, 1993). Allport (1961) described personality as a dynamic system that determines the behavior, feelings, and thoughts of an individual. In our study, we considered the personality dimensions of the Big Five (McCrae et al., 1998). The Big Five includes extraversion, agreeableness, conscientiousness, neuroticism, and openness to experiences. These traits show numerous correlations with media use for the gratification of social needs. For example, extraversion and openness were positively associated with the use of social media (Correa et al., 2010). This association was particularly strong among younger adults. Extroverted people tend to be more loyal to themselves in face-to-face interactions, while neurotic and introverted people found it easier to be themselves in online communication (Amichai-Hamburger et al., 2002). Furthermore, introverts were more likely to dwell on digital communication because they felt less anxious after CMC than after face-to-face interaction (Rice and Markey, 2009). Horzum (2016) found relations between personality traits and differences in motives for social media use. People with a high degree of extraversion used social media for the gratification of meeting new people, socializing as well as informational and educational gratifications. Agreeableness was positively related to gratifications of maintaining existing relationships as well as for informational and educational gratifications. To sum up, previous studies suggest a significant role of the Big Five in CMC during lockdowns in the COVID-19 pandemic.

#### Fear of Missing Out

Fear of missing out represents the fear that interpersonal interactions might be missed. It is a fear of other people are having fun without themselves, and it has been linked to increased social media use (Przybylski et al., 2013). In fact, Blackwell et al. (2017) found that fear of missing out predicted the use of social media. Also, Wang et al. (2018) suggested that people with higher levels of fear of missing out present themselves online to improve their connection with others. Their findings also showed that people with a high (vs. low) need for social connections are more afraid that their friends might have rewarding experiences without them. Through the increased use of social media, the fear of missing out could increase social connections (Roberts and David, 2020) and hence reduce the perceived social isolation during a pandemic. Consequently, if face-to-face contact with other people is lost due to contact restrictions, this fear could be triggered and may lead to more media use.

#### Preference for Solitude

Social isolation and loneliness can influence media use to stay in contact with other people. Because humans are understood as social beings, it is assumed that humans have a general need to spend time with other people (Baumeister and Leary, 1995). Perceived loneliness can be understood as a state characterized by a lack of social interaction (e.g., Storr, 1988; Koch, 1994; Burger, 1995). This state can but does not necessarily have to be accompanied by the physical isolation of the person. Nevertheless, people differ in how they react to the lack of social contacts. These differences can be traced back to inter-individual differences in the preference for solitude (e.g., Storr, 1988; Koch, 1994; Burger, 1995). People who prefer solitude like to be alone and spend more evenings alone at home (Nestler et al., 2011). This preference could also be relevant regarding the use of CMC in order (not) to spend time together with other people in their leisure time. For people who prefer being alone, contact restrictions may be changeless in their leisure activities, so they may be less likely to use CMC as a substitute for social needs or to communicate with others, compared with people who do not prefer solitude.

Given the potential relevance of all these personal characteristics for CMC behavior of people in the context of leisure activities during a nationwide lockdown, we asked:

RQ4: Are personal characteristics of users (age, gender, self-efficacy, state affect, Big Five, fear of missing out, and preference for solitude) related to the number of different leisure activities that people experience with others *via* CMC during the lockdown?

Finally, how people maintain their social contacts during current contact restrictions may have implications for future use of CMC. This is of central importance regarding interaction styles in a post-pandemic era since a pandemic can repeatedly lead to further lockdowns, as happened again in Germany at the end of 2020. According to the U&G approach, it is more likely to choose a medium again, if the medium has already successfully satisfied relevant needs (Palmgreen and Rayburn, 1982). Thus, in addition to personal characteristics, quantity and perceived quality of CMC may be decisive for future use of communication media. We therefore asked the following research question:

RQ5: Are personal characteristics as well as the quantity and perceived quality of current CMC in the context of leisure activities related to motivation of people to continue CMC after the pandemic?

## Materials and Methods

### Participants

The study included a final data set of 679 full-aged German-speaking participants (558 women, 82.2%) with a mean age of 34.15 years (*SD* = 12.73, range = 18–69). Eight participants were previously excluded due to incomplete data sets. The most frequently stated educational attainment was a higher educational entrance qualification (*n* = 210, 30.9%), followed by a degree in master or diploma (*n* = 155, 22.8%), a completed vocational training (*n* = 138, 20.3%), a degree in bachelor (*n* = 112, 16.5%), a secondary school certificate (*n* = 55, 8.1%), and a main school graduation (*n* = 9, 1.3%). Participants lived together with an average of 2.08 people (*SD* = 1.32, range = 0–7). The study ran for 29 days, beginning April 13, 2020, by which time the nationwide lockdown and contact restrictions had already been in effect for 3 weeks.

The participants were recruited through convenience sampling. The link to the study was broadly disseminated *via* mailing lists, social media, and a survey platform of a national journal (Psychologie Heute). Participation in the study was voluntary and no incentives were provided. No identifying data were collected to guarantee anonymity of participants. At the beginning of the study, participants were informed about the purpose of the study, that all data would be processed only for research purposes, that they would remain anonymous, and that they could prematurely abandon the study at any point in time. The participants finally indicated informed consent by clicking a corresponding box.

### Procedure

Participants initially provided their gender, age, the number of people with whom they live together, and their highest educational qualification. Afterward, they were asked several questions about their media use, including contacts with people in leisure time, leisure activities *via* CMC, and quality and quantity of CMC. Then, the participants responded to a series of person-related questions covering self-efficacy, state affect, Big Five personality traits, fear of missing out, and preference for solitude.

### Measures

#### Media Use Variables

Participants reported the number of people they met personally in their leisure time in a normal week before the COVID-19 pandemic and (now) during the nationwide lockdown, excluding people who live in the same household. Then, the participants reported the number of people with whom they have currently contacted *via* CMC in a normal week during the lockdown and their current perceived social closeness to them. Following Lee and Gillath (2016), the latter was measured by a single item “How close do you currently feel to all those people with whom you can currently only communicate *via* media in your leisure time?” (from 1 = “not close at all” to 7 = “very close”).

**Figure 1:**
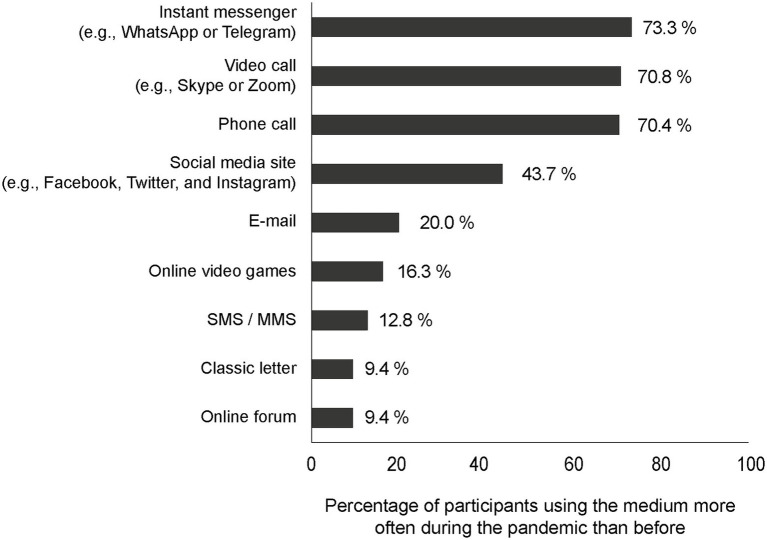
Percentage of participants who reported using communication media more frequently in the context of leisure activities during the first nationwide lockdown than before the pandemic.

Next, participants were presented with a list of nine communication media: classic letter, SMS/MMS, e-mail, phone call, video call (e.g., Skype or Zoom), instant messenger (e.g., WhatsApp or Telegram), social media sites (e.g., Facebook, Twitter, and Instagram), online forums, and online video games. They indicated with “yes” or “no” to which of the listed media they currently use more frequently in their leisure time to communicate with others than before the pandemic (see Figure 1). We chose not to measure the absolute amount of time spent with each medium prior to the pandemic because previous studies have shown that retrospective self-reports of media use are often biased (Collopy, 1996; Araujo et al., 2017). Therefore, we selected a less fine-grained but more reliable measurement.

**Figure 2:**
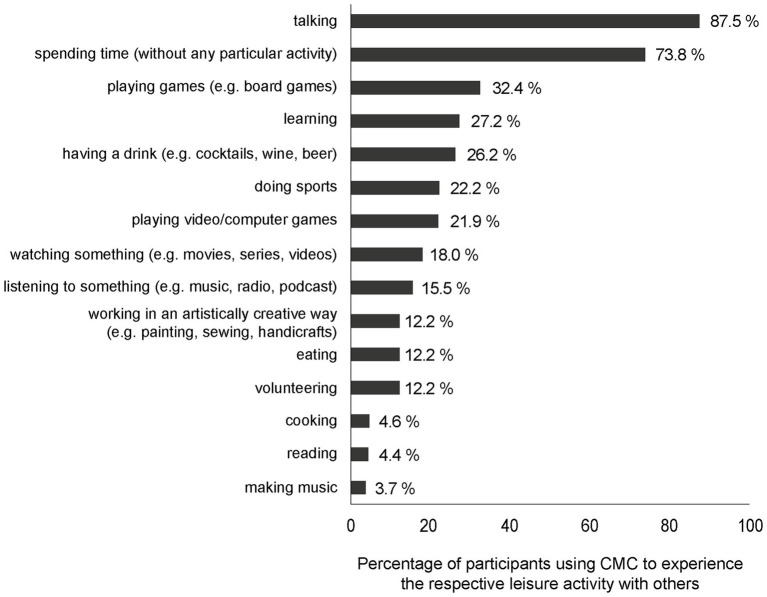
Percentage of participants who reported experiencing certain leisure activities together with others *via* CMC during the first nationwide lockdown.

Then, participants stated (“yes” or “no”) to which of 15 activities they currently experience together with other people in their leisure time *via* CMC (e.g., “I use media to learn together with others” or “I use media to cook together with others.”). A complete list of all activities is presented in Figure 2. A summed value was calculated (from 0 to 15), indicating the number of different leisure activities that people experience with others *via* CMC. This sum value served as a dependent variable in multiple regression analysis in section “Inter-Individual Differences in Media Use for Leisure Activities During Contact Restrictions (RQ4)”.

The perceived quality of CMC in the context of leisure activities was assessed by asking “How satisfied are you currently with the communication you have with other people through media in your leisure time (e.g., smartphone, telephone, PC)?”, using a single-item scale ranging from 1 (“not satisfying at all”) to 7 (“very satisfying”). To measure the quantity of CMC in the context of leisure activities, participants responded to the question “How intensively do you currently communicate with other people by means of media (e.g., smartphone, telephone, PC) in your leisure time?” (from 1 = “not intensively at all” to 7 = “very intensively”). The quality and quantity of CMC were part of a moderated regression analysis in section “Computer-Mediated Communication in Leisure Time During Contact Restrictions (RQ1, RQ2, and RQ3)”. Then, participants rated their motivation to continue CMC with others after the pandemic, even if direct face-to-face contact would be possible again (from 1 = “very little motivated” to 7 = “very strongly motivated”). This variable served as the dependent variable in multiple regression analysis in section “Motivation for Computer-Mediated Communication After Contact Restrictions (RQ5)”.

#### Person-Related Variables

##### General Self-Efficacy

General self-efficacy was measured by using the short scale of Beierlein et al. (2012), covering three items (“In difficult situations I can rely on my abilities.”, “I am able to solve most problems on my own.”, “I can usually solve even challenging and complex tasks well.”, Cronbach’s *α* = 0.83). The scale uses a five-point format (from 1 = “not true at all” to 5 = “completely true”). According to Schwarzer and Jerusalem (1999), the original 10-item scale has a reliability of *α* = 0.92, so using the short scale is associated with an acceptable loss of reliability.

##### Positive and Negative State Affect

The German version of the positive and negative state affect (PANAS; Watson et al., 1988) adapted from Krohne et al. (1996) was used to assess positive (*α* = 0.85) and negative affect (*α* = 0.82) of participants experienced in the past few days during the first nationwide lockdown. This scale consists of 20 emotion-laden adjectives (e.g., active, interested, strong, guilty, and nervous) and the possible answers range from 1 to 5 (1 = “not at all”, 2 = “a little”, 3 = “somewhat”, 4 = “considerably”, and 5 = “extremely”).

##### Big Five Personality Traits

The BFI-K (Rammstedt and John, 2005) was used to measure extraversion of participants (four items, e.g., “I go out of myself, I am sociable.”, *α* = 0.80), neuroticism (four items, e.g., “I get easily depressed, dejected.”, *α* = 0.79), agreeableness (four items, e.g., “I easily trust others, believe in the good in people.”, *α* = 0.64), conscientiousness (four items, e.g., “I complete tasks thoroughly.”, *α* = 0.65) and openness to experiences (five items, e.g., “I am interested in many things.”, *α* = 0.74). The answer options are “very incorrect”, “rather incorrect”, “neither nor”, “rather correct” and “very correct”. The scale does not include numerical markers.

##### Fear of Missing Out

Fear of missing out was measured by a scale of Przybylski et al. (2013), which was translated into the German language for the present study (translate-translate back method). Item wording was also guided by a previous German version developed by Spitzer (2015). The scale comprises 10 items (e.g., “I fear others have more rewarding experiences than me.” or “I get anxious when I do not know what my friends are up to.”, *α* = 0.77). The scale ranges from 1 (“does not apply to me at all”) to 5 (“applies extremely well to me”). No verbal markers were presented between the endpoints of the scale.

##### Preference for Solitude

A German scale measuring preference of one for solitude was used which is composed of 10 items (Nestler et al., 2011). The scale (from 1 = “does not apply at all” to 6 = “very much applies”) covers two components, namely the need to be alone and the joy of being alone. These two subscales were aggregated to get the overall preference for solitude (*α* = 0.84).

## Results

We ran all analyses with SPSS 27. Before the analyses were calculated, all statistical assumptions were checked. This was particularly relevant for the multiple regression models (cf. Poole and O’Farrell, 1971) to assess the robustness of the results. We did not find outliers, multicollinearity, or autocorrelation in any of the three regression models. Furthermore, linearity and normality were given in all models. However, slight hints for heteroscedasticity were found, so we used bootstrapping method for statistical testing (5,000 iterations).

First, we checked whether the participants complied with the requirements of government to reduce direct face-to-face contact with other people. As expected, participants had less direct face-to-face contact with others (*M* = 1.32, *SD* = 2.56) in their leisure time during the first nationwide lockdown compared to the number of direct contacts they had before the pandemic [(*M* = 9.17, *SD* = 13.09), *t*(678) = 15.84, *p* < 0.001, *d* = 0.61]. Hence, the participants had very limited face-to-face contact at the time of the survey.

### Computer-Mediated Communication in Leisure Time During Contact Restrictions (RQ1, RQ2, and RQ3)

We analyzed how many participants used the listed media more often for communication purposes during leisure time than before the pandemic (RQ1). As shown in Figure 1, especially synchronous media formats were used more frequently. Instant messengers were used more frequently by 73.3% of participants, video calls by 70.8%, and phone calls by 70.4%.

Further, we analyzed which leisure activities people experience with others *via* CMC during the lockdown (RQ2) and as shown in Figure 2, participants experienced a wide variety of leisure activities. Most participants used CMC to talk to other people or to simply spend time together without any particular activity. The least common use of CMC was to listen to music, to read, and to cook together with others. On average, participants used CMC for 3.74 (*SD* = 2.09) of the 15 listed activities in their leisure time. The number of different leisure activities that participants experienced with others *via* CMC was further scrutinized in RQ4 in section “Inter-Individual Differences in Media Use for Leisure Activities During Contact Restrictions (RQ4)”.

In the next step, a moderated regression (bootstrapping with 5,000 iterations) was calculated. We investigated the relationship between the perceived quality of CMC in the context of leisure activities and the perceived social closeness with other people, and whether this relationship was moderated by the quantity of CMC in the context of leisure activities (RQ3). To avoid problems of multicollinearity, the independent and moderator variables were initially centered and then included in the model together with their interaction term. Perceived social closeness served as the dependent variable. The regression model was significant, *F*(3, 675) = 64.02, *p* < 0.001, with an *R*^2^ of 0.22. Both the perceived quality (β = 0.438, *p* < 0.001) and the quantity (β = 0.118, *p* = 0.006) of CMC showed a significant relation to perceived social closeness to other people. However, the interaction term was not significant (β = −0.008, *p* = 0.855). Hence, the perceived quality of CMC and perceived social closeness to others were positively related, whereas the quantity of CMC did not moderate this relationship but showed itself a significant relation to perceived social closeness.

### Inter-Individual Differences in Media Use for Leisure Activities During Contact Restrictions (RQ4)

A multiple regression analysis was run to investigate the relationship between personal characteristics of users and the number of different leisure activities they experience with others *via* CMC during the lockdown (RQ4). Two participants (0.3%) reported their gender as “diverse” and were excluded from the following analyses as this was an insufficient subsample for the corresponding statistical analysis. In summary, the correlations between the independent variables were rather low with few exceptions, as shown in Table 1. The highest (positive) correlation was between neuroticism and negative affect (*r* = 0.51). The reported quantity and perceived quality of CMC showed only a few weak correlations with personal characteristics.

**Table 1:** Descriptive statistics and bivariate correlations (Pearson *r* and two-tailed *p*-value) among all variables included in regression models.

		*M*	*SD*	Correlations															
				1.	2.	3.	4.	5.	6.	7.	8.	9.	10.	11.	12.	13.	14.	15.	16.
1.	Age	34.16	12.75																
2.	Gender	−	−	0.01															
3.	Self-efficacy	4.07	0.66	0.10^∗^	−0.00														
4.	Positive affect	2.79	0.69	0.04	−0.02	0.43^∗∗∗^													
5.	Negative affect	1.94	0.63	−0.04	0.08^∗^	−0.32^∗∗∗^	−0.34^∗∗∗^												
6.	Extraversion	3.51	0.88	0.06	0.15^∗∗∗^	0.22^∗∗∗^	0.21^∗∗∗^	−0.01											
7.	Neuroticism	2.91	0.94	−0.15^∗∗∗^	0.14^∗∗∗^	−0.46^∗∗∗^	−0.39^∗∗∗^	0.51^∗∗∗^	−0.24^∗∗∗^										
8.	Agreeableness	3.08	0.81	0.13^∗∗^	0.07	0.08^∗^	0.15^∗∗∗^	−0.13^∗∗^	0.20^∗∗∗^	−0.21^∗∗∗^									
9.	Conscientiousness	3.67	0.66	0.10^∗∗^	0.18^∗∗∗^	0.40^∗∗∗^	0.33^∗∗∗^	−0.14^∗∗∗^	0.25^∗∗∗^	−0.21^∗∗∗^	0.13^∗∗^								
10.	Openness	3.74	0.76	0.05	0.06	0.12^∗∗^	0.15^∗∗∗^	0.02	0.10^∗∗^	0.04	0.08^∗^	0.03							
11.	Fear of missing out	2.48	0.67	−0.29^∗∗∗^	0.03	−0.29^∗∗∗^	−0.19^∗∗∗^	0.27^∗∗∗^	−0.05	0.40^∗∗∗^	−0.12^∗∗^	−0.15^∗∗∗^	−0.04						
12.	Preference for solitude	3.85	0.90	−0.04	0.06	0.01	−0.02	0.07	−0.35^∗∗∗^	0.16^∗∗∗^	−0.19^∗∗∗^	−0.10^∗∗^	0.19^∗∗∗^	−0.08^∗^					
13.	Quality of CMC	4.09	1.50	0.04	−0.04	0.16^∗∗∗^	0.26^∗∗∗^	−0.21^∗∗∗^	−0.05	−0.13^∗∗^	0.06	0.03	0.11^∗∗^	−0.11^∗∗^	0.08^∗^				
14.	Quantity of CMC	5.23	1.32	−0.02	0.04	0.01	0.05	0.09∗	0.18^∗∗∗^	0.04	0.02	0.04	0.02	0.13^∗^	−0.14^∗∗∗^	0.14^∗∗∗^			
15.	Perceived social closeness	4.00	1.41	0.07	0.04	0.19^∗∗∗^	0.20^∗∗∗^	−0.18^∗∗∗^	0.10^∗^	−0.10^∗^	0.10^∗∗^	0.07	0.12^∗∗^	−0.12^∗∗^	0.06	0.46^∗∗∗^	0.18^∗∗∗^		
16.	Number of different leisure activities *via* CMC	3.74	2.09	−0.33^∗∗∗^	−0.07	0.01	0.10^∗^	−0.00	0.15^∗∗∗^	−0.01	0.04	−0.05	0.11^∗∗^	0.17^∗∗∗^	−0.11^∗∗^	0.09∗	0.33^∗∗∗^	0.09^∗^	
17.	Motivation to continue CMC after the pandemic	3.59	1.59	−0.03	−0.08^∗^	−0.00	0.12^∗∗^	−0.02	−0.19^∗∗∗^	0.05	0.04	−0.05	0.16^∗∗∗^	0.05	0.16^∗∗∗^	0.49^∗∗∗^	0.12^∗∗^	0.35^∗∗∗^	0.12^∗∗^

All results are based on *n* = 677.

∗∗∗ *p* < 0.001;

∗∗ *p* < 0.01;

∗ *p* < 0.05.

Gender was dummy-coded (0 = male and 1 = female).

Table 2 shows the results of the multiple regression analysis. The number of different leisure activities experienced with others *via* CMC served as the dependent variable. The model explained 18.5% of variance, *F*(12, 664) = 12.52, *p* < 0.001. Age (β = −0.319, *p* < 0.001) was the most important independent variable and was negatively related to the average number of different leisure activities experienced with others *via* CMC. Gender (β = −0.081, *p* = 0.038) also showed a significant relationship with the dependent variable, whereby male experienced more leisure activities with others *via* CMC than women. Regarding the Big Five personality traits, only extraversion (β = 0.124, *p* = 0.003) and openness to experiences (β = 0.130, *p* < 0.001) showed a significantly positive relation to the number of different leisure activities *via* CMC. Positive affect (β = 0.085, *p* = 0.042) and fear of missing out (β = 0.106, *p* = 0.009) both showed a positive relationship to the dependent variable. In contrast, preference for solitude showed a negative relation (β = −0.083, *p* = 0.050). Importantly, the results of the multiple regression are completely consistent with the bivariate correlations between independent variables and the dependent variable (see Table 1).

**Table 2:** Results of the multiple regression analyses.

Independent variable	Number of different leisure activities *via* CMC (*R*^2^ = 0.185, *p* < 0.001)			Motivation to continue CMC after the pandemic (*R*^2^ = 0.315, *p* < 0.001)		
	*B*^b^	β	*p*^c^	*B*^b^	β	*p*^c^
Age	−0.05 [−0.06, −0.04]	−0.319	<0.001	−0.00 [−0.01, 0.01]	−0.017	0.608
Gender^a^	−0.44 [−0.86, −0.01]	−0.081	0.038	−0.28 [−0.59, 0.04]	−0.068	0.071
Self-efficacy	0.02 [−0.25, 0.30]	0.006	0.888	−0.10 [−0.29, 0.10]	−0.040	0.354
Positive affect	0.26 [0.01, 0.51]	0.085	0.042	0.13 [−0.05, 0.31]	0.057	0.156
Negative affect	0.03 [−0.26, 0.30]	0.010	0.818	0.13 [−0.07, 0.33]	0.052	0.199
Extraversion	0.30 [0.10, 0.48]	0.124	0.003	−0.30 [−0.44, −0.16]	−0.165	<0.001
Neuroticism	−0.06 [−0.29, 0.17]	−0.027	0.583	0.03 [−0.12, 0.18]	0.015	0.737
Agreeableness	0.11 [−0.10, 0.31]	0.042	0.294	0.13 [0.00, 0.27]	0.069	0.053
Conscientiousness	−0.23 [−0.47, 0.02]	−0.072	0.076	0.01 [−0.19, 0.20]	0.004	0.922
Openness	0.36 [0.17, 0.55]	0.130	<0.001	0.23 [0.09, 0.37]	0.110	0.002
Fear of missing out	0.33 [0.08, 0.58]	0.106	0.009	0.18 [−0.01, 0.35]	0.075	0.059
Preference for solitude	−0.19 [−0.38, −0.01]	−0.083	0.050	0.12 [−0.01, 0.25]	0.070	0.069
Quality of CMC	N/A^d^	N/A^d^	N/A^d^	0.49 [0.41, 0.57]	0.463	<0.001
Quantity of CMC	N/A^d^	N/A^d^	N/A^d^	0.09 [0.01, 0.18]	0.076	0.038

a 0 = male and 1 = female.

b *B* values represent unstandardized regression coefficients and its 95% CI (bias-corrected and accelerated method).

c *p* values are based on bootstrapping with 5,000 iterations.

d Not applicable.

### Motivation for Computer-Mediated Communication After Contact Restrictions (RQ5)

In the final regression analysis, motivation of the participants to continue CMC after the pandemic served as a dependent variable (RQ5). In addition to the set of personal characteristics already used as independent variables in the first regression model in section “Inter-Individual Differences in Media Use for Leisure Activities During Contact Restrictions (RQ4)”, we added the quality and quantity of current CMC in the context of leisure activities. Overall, the model explained 31.5% of variance, *F*(14, 662) = 21.77, *p* < 0.001. However, while some independent variables nearly missed the significance level, only four independent variables showed a significant relation to the dependent variable (Table 2). Two of these variables belonged to the Big Five: Extraversion (β = −0.165, *p* < 0.001) showed a negative relation and openness to experiences (β = 0.110, *p* = 0.002) showed a positive relation. Also, the reported quantity of CMC in the context of leisure activities during the pandemic showed a positive relation to the motivation to continue CMC after the pandemic (β = 0.076, *p* = 0.038). However, by far the most important independent variable was the (current) perceived quality of CMC in the context of leisure activities (β = 0.463, *p* < 0.001). Hence, the experienced quality of CMC was more important for future media use than the personal characteristics of the media users.

## Discussion

The results of the present study, conducted during the first nationwide lockdown in Germany in spring 2020, showed that people used and created new digital possibilities to stay in contact with others.

First, our data showed that the participants massively restricted their social contacts in their leisure time and thus obeyed the restrictions of the government. This is important to understand the data in its context, where important social needs could no longer be satisfied in face-to-face situations. The percentage of participants who used communication media more frequently in the context of leisure activities during the nationwide lockdown than before differed remarkably across media formats (RQ1). Synchronous communication formats were particularly popular among the participants, especially instant message services. Also, video calls and classic phone calls were used more often by over 70% of all participants. This is consistent with the findings of Gabbiadini et al. (2020), who also found increased use of voice calls during the pandemic in an Italian sample study. Although it is unclear whether synchronicity influences on the perceived social support (Rains and Wright, 2016), our data indicate a clear preference for media that, in principle, enable synchronous communication.

Second, the participants reported performing and experiencing various leisure activities together with others *via* CMC (RQ2). This result is also consistent with the study by Gabbiadini et al. (2020), who found that leisure activities were shifted to the digital sphere, such as watching movies together or playing board games. However, we found a clear preference for low-key activities, such as just talking or simply spending time together. Nevertheless, more complex activities were also performed *via* CMC, such as having a drink together, eating together, and doing sports.

Third, the perceived quality as well as the quantity of CMC in the context of leisure activities during the lockdown showed a significant positive relation to perceived social closeness (RQ3). However, the quantity of CMC was not qualified as a moderator variable in the regression model. Hence, CMC is likely to maintain the perceived social closeness to others, whereby both quality and quantity of the communication process are relevant factors.

Fourth, we investigated the number of leisure activities performed and experienced together with others *via* CMC during the lockdown (RQ4). This number served as the dependent variable in a multiple regression model incorporating personal characteristics of the media users as independent variables. The age of the participant was the most relevant independent variable, with younger people experiencing more leisure activities *via* CMC. This result is in line with previous studies showing that younger people acquire media faster than older people (Czaja et al., 2006). Peer care and communication *via* social media were also found to be more prominent among younger people (Kumar and Lim, 2008; Blackwell et al., 2017). Mixed results were found regarding the relationship between gender and media use in previous research (cf. Ono and Zavodny, 2003; Kimbrough et al., 2013; Özgüven and Mucan, 2013), but in the present study, men reported experiencing more leisure activities with others *via* CMC than women. We also found that higher positive state affect was related to more leisure activities *via* CMC. Negative affect as well as self-efficacy were not significant factors. Regarding the Big Five personality traits, extraversion and openness to experiences were significant positive factors. Correa et al. (2010, p. 247) already found that “extraverted men and women were both likely to be more frequent users of social media tools.”. Similarly, Horzum (2016) reported that people with a high degree of extraversion used social media for the gratification of meeting new people, socializing as well as for informational and educational gratifications. Furthermore, in the definition of openness to new experiences, it is already implied that new things are gladly tried out (McCrae et al., 1998), which is quite consistent in an unprecedented situation of restricted social contracts. Participants with higher (vs. lower) fear of missing out performed and experienced more activities in leisure time with other people *via* CMC. In addition, people with higher fear of missing out felt the need to maintain, particularly, high levels of social activity *via* media. In contrast, and not surprisingly, preference for solitude showed a negative relation to leisure activities *via* CMC. Overall, this set of personal characteristics explained 18.5% of the inter-individual variance in computer-mediated leisure activities during the lockdown. This amount of explained variance remains much room for other potential factors that are not considered here. In more positive terms, given that media literacy, media use habits, available technological infrastructure, etc., presumably also play an important role in this context, it is noteworthy that general personal characteristics that are not specifically related to media use are nonetheless relevant.

Finally, when participants were asked if they would be motivated to continue CMC after the pandemic, we found a different pattern of results. (RQ5): Although nearly one-third of the variance in this motivation (31.5%) could be explained by an extended model. Only two characteristics of the Big Five showed significant relations to use motivation in the future. People with higher (vs. lower) openness to experiences were more motivated to continue CMC even after the pandemic. Extraverted (vs. introverted) people preferred to return to face-to-face communication they usually prefer. Moreover, the current quality and quantity of CMC in the context of leisure activities showed a positive relation to the motivation for CMC after the pandemic. This result is in line with the central assumption of the U&G approach, according to which satisfied expectations predict continued use of a medium (Palmgreen and Rayburn, 1982).

### Limitations

Some limitations of our study are mentioned. First, the present study consisted of self-ratings and did not allow a detailed analysis of the specific style and quality of computer-mediated leisure activities. Second, the participants were recruited through convenience sampling, which means that we may have reached a certain milieu of being not representative for all media users. For example, the mean age of the sample was about 34 years and the study was limited to the status quo in Germany during the first nationwide lockdown in 2020. Indeed, some moderate cross-cultural differences in motives of people for media use and associated gratifications have been reported before the pandemic (e.g., Lee et al., 2014; Sheldon et al., 2017). However, there is no valid reason to believe that German citizens differ substantially from everyone else in their online communications during contact restrictions due to the COVID-19 pandemic, but available technical infrastructure, access to media, and cross-cultural differences regarding preferences for specific leisure activities may be moderating variables. These factors limit the generalizability of our results. Third, the extent and specification of contact restrictions and lockdown conditions may vary across regions and over time. Fourth, during the ongoing pandemic, it is conceivable that people have successively become familiar with the situation, have gained specific media competencies, and purchased technological equipment, facilitating the way of computer-mediated activities with others. Fifth and the last, our data are cross-sectional, correlational data so that no causal conclusions can be drawn.

### Practical Implications

The results of our study have important practical implications. We found that people use digital media in many different ways to stay in contact with their family and friends. Our results indicate that older people perform fewer different activities *via* digital media for this purpose, which could be related to their slower appropriation of digital technologies (Czaja et al., 2006). During contact restrictions, it was recommended not to visit retirement homes, hospitals, and nursing facilities because of the vulnerable people who live there (e.g., Dichter et al., 2020). Due to this situation, older generations could become lonely (Kemptner and Marcus, 2020). CMC could be one possibility to counteract this risk. Therefore, it seems important to support older generations (and other vulnerable people) in their media activities so that they can stay to be in contact with their family and friends *via* media. It should be noted, however, that only around 20% of people over the age of 85 who live alone in Germany have an internet connection to date (Kemptner and Marcus, 2020). The critical infrastructure must therefore be expanded. Especially in Germany, there is still a lack of ultrafast broadband connections (Martins and Wernick, 2021), which can influence on the quality and quantity of media use. We showed that the quality and quantity of CMC were positively related to the perceived social closeness to other people, which underlines the urgency of better internet connectivity and access to digital media. A fast internet connection is particularly important as video calls were one of the most preferred communication formats used by about 70% of participants more frequently during the lockdown than before. Besides, media literacy of users must be enhanced, because many leisure activities migrated into the digital world. Enhancing media literacy among users can increase both knowledge of and criticism toward media, and furthermore, ensure awareness of the influence of media as well as reduce risky or antisocial behaviors (cf. Jeong et al., 2012; Rüth and Kaspar, 2021).

Furthermore, we found that there are inter-individual differences in the extent of leisure activities *via* CMC and motivation to continue CMC even after the pandemic. Different groups of people prefer different ways of communication, for example, extroverts (vs. introverts) prefer face-to-face contact rather than digital communication. On the one hand, these differences have the advantage that like-minded people network with each other and thus form homogeneous groups, which generate a higher sense of equality (Himmelroos et al., 2017). On the other hand, the formation of these homogeneous groups can lead to echo chambers which could enhance polarization (cf. Baumann et al., 2020). In order to prevent such group formation processes, to achieve as much diversity as possible, and to create a satisfying experience with digital media for all users, it seems important to improve the usability of systems. One standard that can be used to judge systems is ISO 9241-11 (1999), which was developed by the International Organization for Standardization and addresses the compatibility of software for the needs of users. It includes important aspects in the design of systems that satisfy individual needs, for example, suitability for the task, self-descriptiveness, conformity with user expectations, and notable suitability for individualization. It seems important to consider the user of digital systems as an active user who selects media entirely according to his or her individual preferences. Our results support the assumptions of U&G in this regard.

## Conclusion

To sum up the results of the present study, we found an important role of digital media in maintaining social contacts. Especially in times of a pandemic, when local lockdowns and contact restrictions repeatedly occur, digital media can help to continue leisure activities with other people and maintain the perceived social closeness to others through CMC. Moreover, personal characteristics, as well as the quantity and quality of current CMC, are associated with the extent to which people perform and experience leisure activities with others *via* digital media. Hence, a more differentiated perspective on the inter-individual differences between media users would be a promising step to create even more satisfying communication formats.

## Data Availability Statement

The raw data supporting the conclusions of this article will be made available by the authors, without undue reservation.

## Ethics Statement

Ethical review and approval was not required for the study on human participants in accordance with the local legislation and institutional requirements. The participants voluntarily participated in this study and indicated informed consent to participate by clicking a corresponding box.

## Author Contributions

JM, KK, and JN developed the study idea, interpreted the results, and wrote the manuscript. JM and KK designed the study and performed the analyses. JM and JN collected the data. KK organized and supervised the data collection. All authors contributed to the article and approved the submitted version.

### Conflict of Interest

The authors declare that the research was conducted in the absence of any commercial or financial relationships that could be construed as a potential conflict of interest.
